# Hierarchical closeness-based properties reveal cancer survivability and biomarker genes in molecular signaling networks

**DOI:** 10.1371/journal.pone.0199109

**Published:** 2018-06-18

**Authors:** Tien-Dzung Tran, Yung-Keun Kwon

**Affiliations:** 1 Complex Systems and Bioinformatics Lab, Hanoi University of Industry, Hanoi, Viet Nam; 2 School of IT Convergence, University of Ulsan, Ulsan, Republic of Korea; University of Ulm, GERMANY

## Abstract

Specific molecular signaling networks underlie different cancer types and quantitative analyses on those cancer networks can provide useful information about cancer treatments. Their structural metrics can reveal survivability of cancer patients and be used to identify biomarker genes for early cancer detection. In this study, we devised a novel structural metric called hierarchical closeness (HC) entropy and found that it was negatively correlated with 5-year survival rates. We also made an interesting observation that a network of higher HC entropy was likely to be more robust against mutations. This finding suggested that cancers of high HC entropy tend to be incurable because their signaling networks are robust to perturbations caused by treatment. We also proposed a novel core identification method based on the reachability factor in the HC measure. The cores were permitted to decompose such that the negative relationship between HC entropy and cancer survival rate was consistently conserved in every core level. Interestingly, we observed that many promising biomarker genes for early cancer detection reside in the innermost core of a signaling network. Taken together, the proposed analyses of the hierarchical structure of cancer signaling networks may be useful in developing future novel cancer treatments.

## Introduction

Cancer is a leading cause of disease worldwide with more than 11 million people diagnosed every year. It is estimated that by 2030 there will be approximately 26 million new cancer cases and 17 million cancer deaths worldwide per year [1]. Cancer is a genetic disease where one or more mutated genes result in abnormal cell proliferation. Early diagnosis and personalized therapy often rely on insights from relevant molecular signaling pathways as well as cancer-related genes. In terms of network dynamics, a signaling network converges on a stable equilibrium state (an ordinary attractor), which corresponds to a normal cellular state, but a genetic mutation may attract a cell to a malignant phenotypic state (a cancer attractor), which eventually results in cancer development [2]. Although a perturbation of a gene or an interaction can be treated to recover the ordinary attractor, the cancer network may be strongly robust to the perturbation. Intriguingly, it has been reported that network robustness is highly related to a variety of structural characteristics, including feedback loops, auto-regulation, feed-forward loops, source—sink gradients, modularity, redundancy, and parallel pathways [3–7]. Therefore, the degree to which the malignant status is maintained against therapeutic perturbations can be related to various structural characteristics in cancer signaling networks. Previous studies have explored this idea [8–10]. In one study, the 5-year survival rates of patients diagnosed with one of 14 different types of cancers were shown to be negatively correlated with the degree of heterogeneous connectivity in all cancer signaling networks except the signaling network underlying prostate cancer [8]. A follow-up study proposed network modularity as a factor in patient survivability and indeed showed that the degree of modularity was negatively correlated with survivability in the same 14 cancers, including prostate cancer [9]. However, network modularity is not an easy-to-compute metric because a non-deterministic algorithm should be used to optimize the metric [11–13]. Taken together, it is more suitable to create a novel metric that can more accurately predict cancer survivability with little computational cost. Thus, we propose a novel metric based on a hierarchical structure.

Our new metric also relates to the core-periphery structural identification of directed networks. It is known that a number of molecular biological networks exhibit a core-periphery structure, wherein a few nodes are highly interconnected and the remaining nodes are loosely connected. Core nodes are identified by pruning underutilized links until core-peripheries emerge [14] and play a differing role from peripheral nodes [15, 16]. For example, in protein contact networks deleterious mutations which may cause cancer are more likely to be distributed near core nodes than peripheral nodes [17]. For instance, some prognostic genes of hepatocellular carcinoma, such as *B1* and *Sec62*, often reside at the core of gene co-expression networks [18]. Among the most recently proposed core identification methods [14, 19–21], *K-core* [22], which is based on the connectivity of a node, has been the most widely used method and was used to show that the core captures the most important characteristics of a network [23–25]. For example, biomarkers were found inside the core area of a co-expression network associated with liver cirrhosis [26] and a protein-protein interaction network associated with hepatocellular carcinoma [27]. However, *K-core* identification is not effective in the analysis of directed networks because the direction of interactions is not considered in the analysis. Therefore, a core identification method adequate for directed networks such as molecular signaling networks is required.

Hierarchical closeness (HC) is a heterogeneous network centrality measure proposed in our previous study [28]. We extend on this previous study in two ways. First, we suggest that HC values of entropy are a novel structural measure to evaluate cancer survivability, and we found that the HC entropy values were negatively correlated with patient 5-year cancer survival rates in 16 types of cancers. This relationship was more clear than in cases using degree entropy [8] and modularity [9]. We also found that HC entropy values were positively correlated with network robustness when extensive simulations based on random Boolean networks were utilized. Since network robustness represents how likely a network is to maintain an equilibrium state against perturbations, this result explains why cancers with relatively high HC entropy values tend to be incurable. Second, we exploit the reachability property to profile the core-periphery structure of directed networks and propose a novel network decomposition called *R-core*. Interestingly, the core area identified by *R-core* maintained the negative relationship of HC entropy to the cancer survival rate whereas the core area identified by the *K-core* decomposition did not. Furthermore, we discovered that many candidate cancer biomarker genes reside in the innermost core area identified by *R-core*, and most of those candidate biomarker genes were validated empirically. This demonstrates that our method identified the functional central region of a directed molecular network.

## Materials and methods

### Cancer signaling networks and 5-year survival statistics

We used 16 pathways downloaded from the Kyoto Encyclopedia of Genes and Genomes (KEGG) (www.genome.jp/kegg) by adding two additional pathways of the Gastro and the Breast cancers to those in the previous studies [8, 9]. KEGG includes comprehensive pathways manually derived from textbooks, literature, other databases, and expert knowledge and provides consensus information regarding the “cancer site” which refers to the tissue or cell type of the primary tumor. We did not consider other pathway databases such as BioCyc (www.biocyc.org), Reactome (www.reactome.org), and BioGRID (www.thebiogrid.org) for analysis since they do not include pathways corresponding to a specific cancer site.

Each cancer pathway is represented by a network, wherein a node and an edge correspond to a protein and an interaction between proteins, respectively. In the network, directed edges represent activation, inhibition, expression, indirect-effect, interaction via compound, missing-interaction, and phosphorylation whereas undirected edges represent protein-protein interactions such as binding/association and dissociation. All types of interactions were included in structrual analysis about HC entropy and K-core/R-core decomposition by interpreting undirected edges as bi-directional interactions. However, we note that only activation and inhibition types of interactions were considered for network robustness estimation as done by previous studies [29, 30]. It should be also noted that the original KGML (KEGG Markup Language) files downloaded from KEGG pathway database were not consistent to the static pathway map image, and we corrected this inconsistency by using the Cytoscape plug-in KEGGParser [31]. The patient 5-year survival rates for every cancer type were obtained from the Surveillance Epidemiology and End Results (SEER) Program database (www.seer.cancer.gov), which is a resource for epidemiological data compiled by the U.S. National Cancer Institute (www.cancer.gov).

### Hierarchical-closeness (HC) entropy

There have been a number of different entropy measures proposed for general network analyses [32–36]. In systems biology, some network entropies were used to identify dynamic changes in time course differentiation data and to predict higher levels of cellular plasticity in cancer stem cell populations [37]. Among them, degree entropy was proposed to be a significant cancer system property in undirected networks such as in a protein-protein interaction network [38]. The Shannon entropy of a discrete random variable *X*, *H*(*X*), is defined as

$$H(X)=-\sum_{x\in X}p(x){\text{log}}_{2}p(x)\;$$ (1)

where *p*(*x*) is the probability mass function of *X*. The degree entropy of a network is obtained by letting *X* represent degree values of the network, where each value *x* ∈ *X* is the number of interactions incident to a node. In this study, we derived another network entropy, HC entropy, by letting *X* represent HC values of the network, where each value *x* ∈ *X* is the HC value of a node. The HC value of a node *v*, *HC*(*v*), was defined previously [28] by combining reachability and closeness as

HC(v)=R(v)+C(v) (2)

where *R*(*v*) represents the reachability value of node *v*, which is the number of nodes in the network that can be reachable from node *v*, and *C*(*v*) is the closeness centrality normalized into [0, 1] [39]. *C*(*v*) is defined as

$$C(v)=\frac{1}{|V|-1}\sum_{w\in V\backslash \{v\}}^{}\frac{1}{d(v,w)}$$ (3)

where |*V*| is the number of nodes, and *d*(*v*, *w*) is the distance of the shortest path, if any, from *v* to *w*; otherwise, *d*(*v*, *w*) is specified as an infinite value. Considering that *R*(*v*) is an integer and *C*(*v*) ∈ [0, 1), the definition of *HC*(*v*) implies that all nodes *v* ∈ *V* are first grouped by *R*(*v*) and the nodes of a same group are further grouped by *C*(*v*). Based on (1), (2), and (3), we computed HC entropies of 16 cancer pathways.

### K-core and R-core decomposition

**Fig 1:**
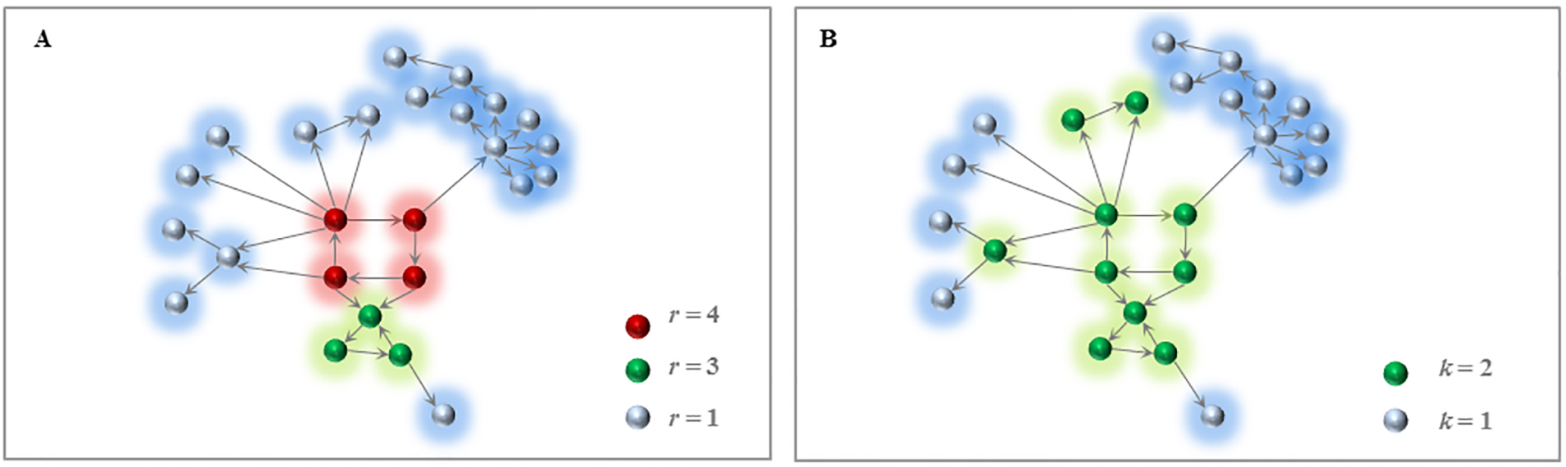
An example of *R-core* and *K-core* decompositions. A directed network with 25 nodes and 29 interactions is given. **(A)** Result of the *R-core* decomposition. Three *r-shells* (*r* = 1, 3, and 4; light blue, green, and red, respectively) were identified. **(B)** Result of the *K-core* decomposition. Two *k-shells* (*k* = 1 and 2; light blue and green, respectively) were identified.

In general, networks can be decomposed into a dense core and a loosely connected periphery by utilizing a network decomposition method. *K-core* decomposition based on the node degree is often used [40] to identify particular subsets of a network, called *k*-*cores* (*k* ≥ 1), where *k* denotes a core level. A *k*-*core* of a network *G* is composed of a subset of nodes in a network *G*, which is obtained by the following pruning rule. Given a network, all the nodes with a degree < *k* are removed, along with their incident interactions, from the network. This removal process is repeated until the degree of every node in the remaining network is ≥ *k*. The *k*-*core* denotes the remaining set of nodes, and hence it holds that *k*_1_-*core* is a subset of *k*_2_-*core* if *k*_1_ ≥ *k*_2_. In this study, we suggest another network decomposition, *R-core*, which is based on the reachability value *R*(*v*). It employs the same pruning rule as the *K-core* decomposition method except *R*(*v*) is used instead of the node degree. In other words, all nodes with *R*(*v*) < *r* and their incident interactions are removed at every pruning step. As a result, *R-core* decomposes a directed network into sub-networks called *r-cores*. By the decomposition definition, *k*- and *r*-*cores* represent a greater inner core as the core level value increases. Furthermore, a *k-shell* (or *r-shell*) is defined as a set of nodes that belong to the *k-core* (or *r-core*) but not to (*k* + 1)-*core* (or (*r* + 1)-*core*). An example of these network decompositions is provided (Fig 1). For convenience, the *periphery* and the *outermost core* denote the shell and the core with the lowest level, respectively. In addition, *1-* and *2-innermost core* denote cores with the first and the second highest level, respectively.

### Network robustness

To evaluate network robustness of both cancer pathways and random networks, we employed a Boolean network model, which has been extensively used to investigate the dynamics of various signaling networks [41–44]. A signaling network is represented by a directed graph *G*(*V*, *A*) where *V* = {*v*_1_, *v*_2_, …, *v*_*n*_}is a set of Boolean variables and *A* is a set of the directed interactions. Each *v*_*i*_ has a value of 1 (‘on’) or 0(‘off’) which represents activated or inactivated statuses, respectively, and the state of *v*_*i*_ should be updated by a corresponding logical function *f*_*i*_. In this study, either a logical conjunction or a disjunction is randomly selected for each *f*_*i*_ uniformly at random. For example, if a Boolean variable *v* has activation relationships with *v*_1_ and *v*_2_, and an inhibitory relationship with *v*_3_, then the respective conjunction and disjunction update rules are $v\left(t+1\right)=\wedge (v_{1}\left(t\right),v_{2}\left(t\right),{\bar{v}}_{3}\left(t\right))$ and $v\left(t+1\right)=\vee (v_{1}\left(t\right),v_{2}\left(t\right),{\bar{v}}_{3}\left(t\right))$. In the case of the conjunction, the value of *v* at time *t* + 1 is 1 only if the values of *v*_1_, *v*_2_, and *v*_3_ at time *t* are 1, 1, and 0, respectively. On the contrary, in the case of the disjunction, the value of *v* at time *t* + 1 is 1 if either *v*_1_(*t*) = 1, *v*_2_(*t*) = 1, or *v*_3_(*t*) = 0 holds. In addition, the states of all genes are synchronously updated. Then a network state defined as *s*(*t*) = (*v*_1_(*t*), *v*_2_(*t*),…, *v*_*n*_(*t*)) at time *t* transits to the next state *s*(*t* + 1) according to a set of update rules defined as *F* = {*f*_1_, *f*_2_,…, *f*_*n*_}(i.e., *s*(*t* + 1) = *F*(*s*(*t*)). The network eventually converges to a fixed state, or a limit-cycle attractor. We denote the attractor starting from state *s*(*t*) as 〈*s*(*t*)〉. The network is called robust to a perturbation at *v* if 〈*s*〉 equals to $\langle s_{\bar{v}}\rangle$ where $\bar{v}\left(=\neg v\right)$ indicates the state perturbation of *s* subject to *v*. This robustness concept has been widely used [45–47]. Specifically, the robustness of a network *γ*(*G*) is defined as follows:

$$\gamma \left(G\right)=\frac{1}{n\cdot |S|}\sum_{v\in V}\sum_{s\in S}I(\left\langle s\right\rangle =\left\langle s_{\bar{v}}\right\rangle )$$ (4)

where *S* is a set of whole network states (here, |*S*| = 2^*n*^), and *I*(⋅) is an indicator function, it outputs 1 if *I*(*true*) or 0 otherwise.

## Results

### HC entropy indicates cancer 5-year survival rates

**Fig 2:**
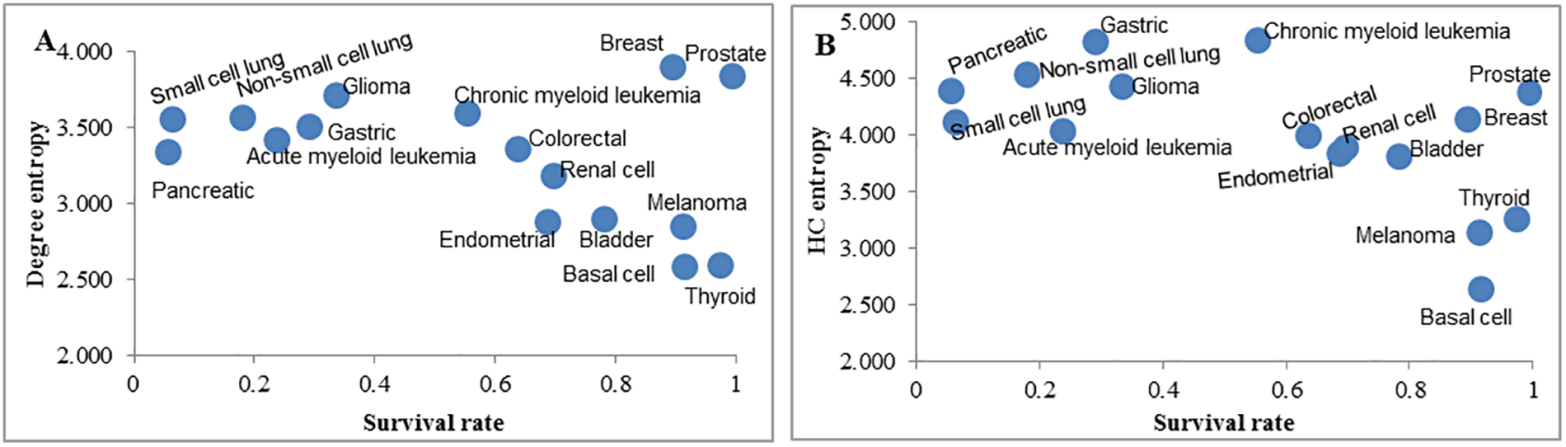
The correlation of degree entropy and HC entropy, respectively, with patient 5-year survival rates for 16 cancer signaling networks. **(A)** Result of degree entropy. A significant correlation is not observed (Spearman’s rank correlation coefficient *R*_*s*_ = −0.294, *P* = 0.255). **(B)** Result of HC entropy. A significant negative correlation is indicated (Spearman’s rank correlation coefficient *R*_*s*_ = −0.591, *P* = 0.022).

**Fig 3:**
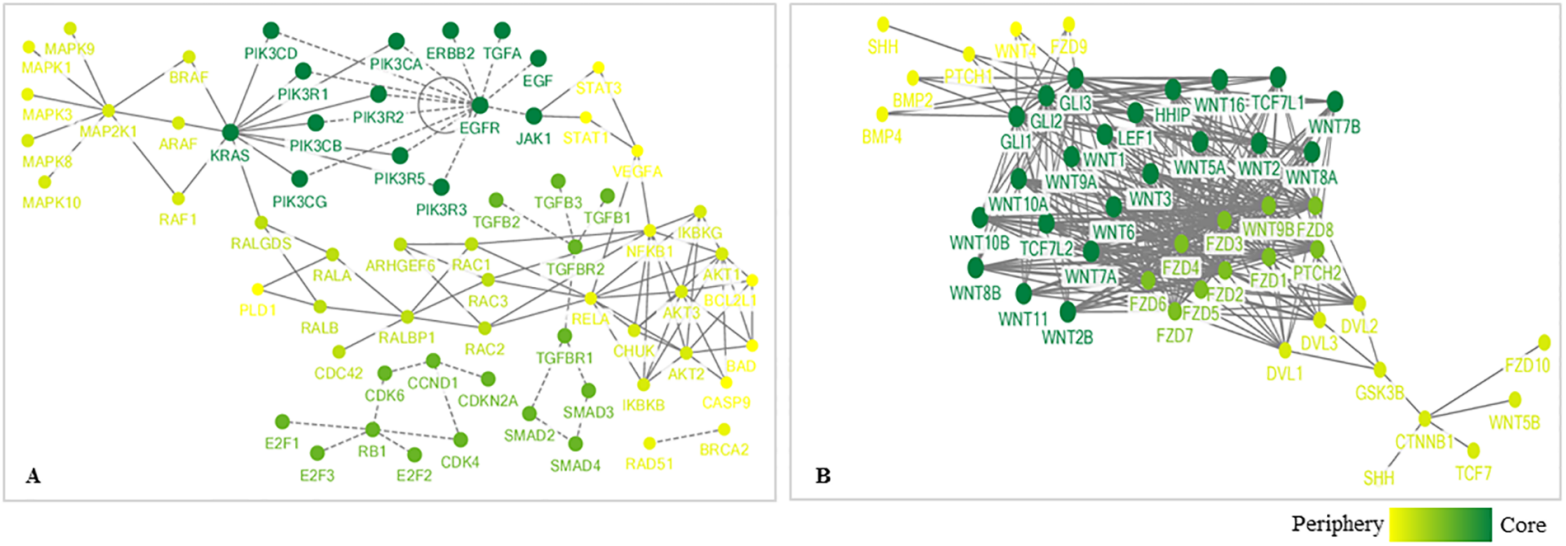
Topological visualization of two cancer signaling pathways with different HC entropy values. **(A)** A pancreatic cancer signaling network shows relatively high HC entropy (4.40) and a low patient 5-year survival rate (0.055). **(B)** A basal cell carcinoma signaling network shows relatively low HC entropy (2.65) and a high patient 5-year survival rate (0.914). The distribution of HC values is more heterogeneous in pancreatic cancer than in basal cell carcinoma networks (see S1 Fig). Dashed and solid lines represent undirected and directed interactions, respectively. The core-periphery structures are profiled by *R-core* decomposition. Dark green and light yellow circles represent nodes in shells of a higher and lower level, respectively. Node labels represent NCBI gene symbols.

A previous study found that the degree entropy of cancer signaling networks correlates to cancer patient survivability in all cases analyzed with the exception of prostate cancer [8]. Another previous finding showed that hierarchical closeness can effectively predict genes more susceptible to mutations [28]. These findings led us to investigate the correlation between patient 5-year survival rates and two entropy measures, degree entropy and HC entropy, of 16 cancer signaling networks (Fig 2). We observed a non-significant negative correlation between the survival rate and degree entropy (Fig 2A; Spearman’s rank correlation coefficient *R*_*s*_ = −0.294, *P* = 0.255), which corroborates previous data [8]. However, HC entropy showed a significant negative relationship (Fig 2B; Spearman’s rank correlation coefficient *R*_*s*_ = −0.591, *P* = 0.022). This comparison suggests that HC entropy is a more accurate measure of 5-year cancer survival rates. Topologies of two example cancer signaling pathways are also shown (Fig 3). Pancreatic cancer has a relatively high HC entropy (4.40) and a relatively low 5-year survival rate (0.055), while basal cell carcinoma has a relatively low HC entropy (2.65) and a relatively high 5-year survival rate (0.914). The nodes of pancreatic cancer signaling pathway tend to be more dispersed than the nodes of the basal cell carcinoma signaling pathway, which illustrates different HC entropies.

### HC entropy reflects robustness of cancer signaling networks

**Fig 4:**
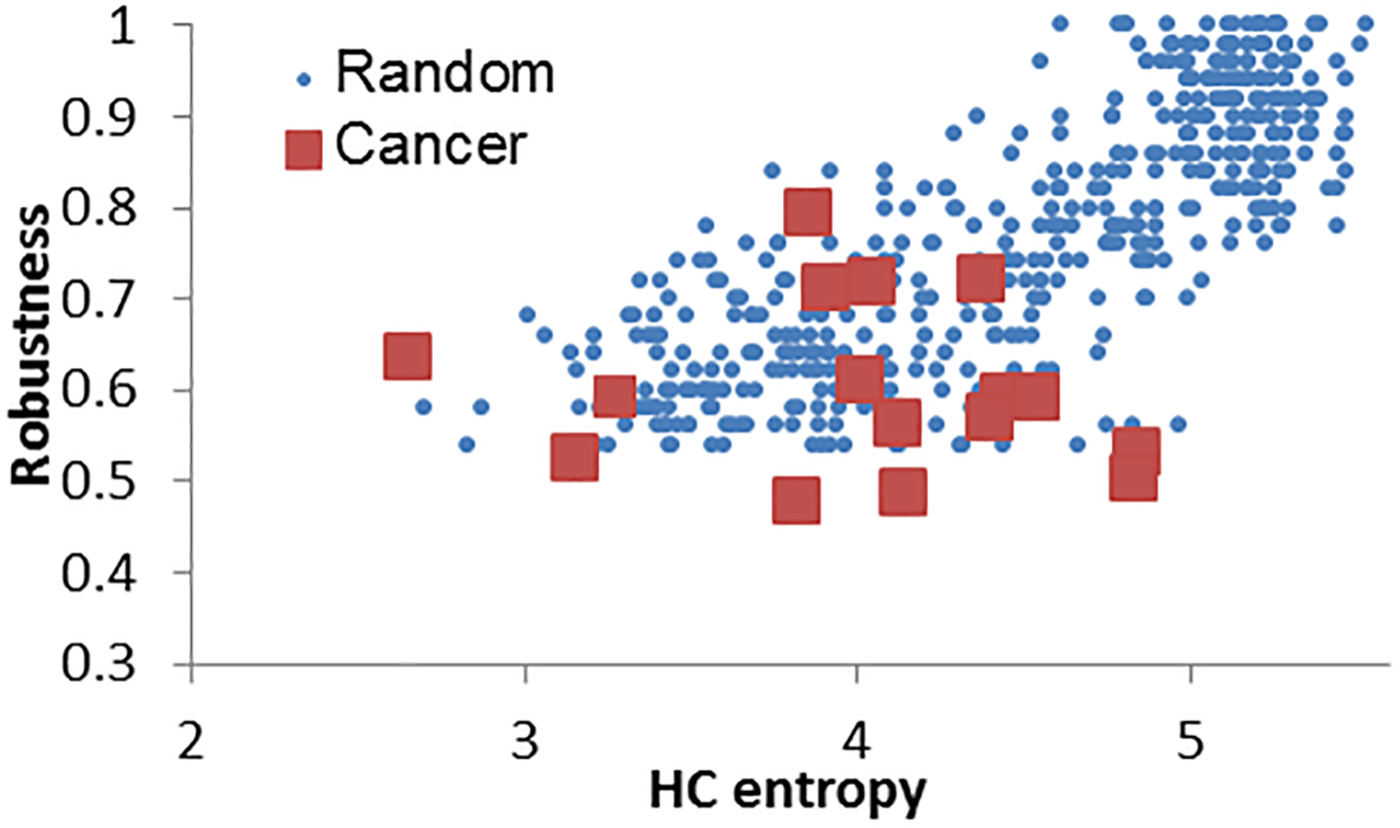
The positive correlation between HC entropy and network robustness in 16 cancer signaling pathways and in random networks. We first constructed a pool of 80000 random networks by shuffling each cancer signaling pathway 5000 times. We considered 24 equal-sized bins by the robustness values ranged from 0.52 to 1 by 0.02, and chose 20 random networks out of the pool for each bin. As a result, we examined HC and robustness values of 480 random networks. The Spearman’s rank correlation coefficient between HC entropy and network robustness in the random networks was significantly positive (*R*_*s*_ = 0.799 with P < 0.0001). The squares represent the results of the 16 cancer signaling networks.

In the previous section, we showed that HC entropy of cancer signaling networks is negatively correlated with cancer survivability. Next, we assumed that HC entropy could be represented by how much a signaling network is robust to mutations and therefore built a Boolean network model to investigate the relationship between HC entropy and network robustness [7, 28]. To do this, we generated random networks using a previous shuffling method [48, 49], which iteratively chooses a pair of interactions (*v*_*a*_, *v*_*b*_) and (*v*_*c*_, *v*_*d*_) uniformly at random and replaces them with a pair of new interactions (*v*_*a*_, *v*_*d*_) and (*v*_*c*_, *v*_*b*_). We note that in-/out-degree distributions of the original network are preserved whereas the reachability distribution is not in the resultant random network. We constructed a pool of 80000 random networks by shuffling each of 16 cancer signaling pathways 5000 trials. For unbiased analysis in the distribution of robustness, we considered 24 equal-sized bins by the robustness values ranged from 0.52 to 1 by 0.02, and chose 20 networks out of the random network pool for every bin. As a result, we investigated the HC entropy and the network robustness values of 480 random networks. As shown in Fig 4, the robustness was positively correlated with the HC entropy in the random networks (Spearman’s rank correlation coefficient *R*_*s*_ = 0.799, P <0.0001). In other words, we observed that the network robustness increased as the heterogeneity of HC values increased. This finding supports the hypothesis that a cancer with a high HC entropy value is likely to be incurable because the corresponding signaling network tends to be robust to therapeutic perturbations. This result is also related to previous observations that showed highly modularized biological networks are sensitive to perturbations because distributions of HC values in these networks are likely to be relatively uniform [7, 50]. Taken together, HC entropy is an interesting architectural characteristic of cancer signaling networks that can indicate network robustness as well as predict patient cancer survival rates.

### The core of cancer signaling networks

**Fig 5:**
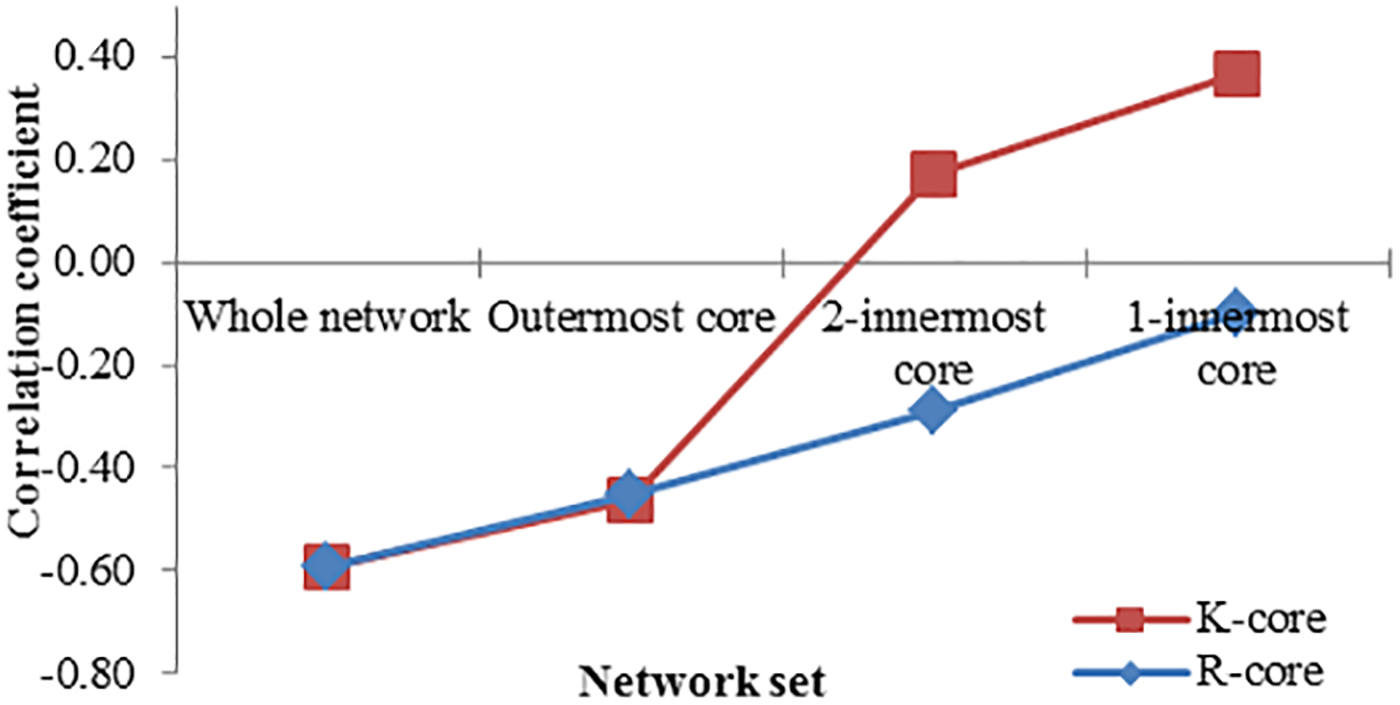
Comparisons of the negative correlation preservation (5-year survival rate, HC entropy) between *K-core* and *R-core* decompositions over 16 cancer signaling networks. Each cancer signaling network was decomposed into three core levels by *R-core* and *K-core* decompositions: *outermost*, *2-innermost*, and *1-innermost* (see Materials and methods for definitions). The correlation coefficient between the 5-year survival rate and the HC entropy of the nodes belonging to each core was respectively compared to that of whole network (−0.591). All the differences between the correlation coefficients of three cores by *R-core* decomposition and that of whole network were not significant (all P values > 0.05). On the other hand, the differences between the correlation coefficients of *2-innermost* and *1-innermost* cores by the *K-core* decomposition and that of whole network were significant (*P* = 0.03 and 0.007 in the case of *2-innermost* and *1-innermost cores*, respectively).

It is important to profile the network core-periphery structure because the core can contain the properties of the whole system. To this point, we compared *K-core* and *R-core* decompositions by determining whether the negative relationship between HC entropy and the 5-year survivor rate was consistently observed within the identified cores. We divided the nodes in the 16 cancer networks into three core levels as determined by either the *K-core* or *R-core* decompositions: *outermost*, *2-innermost*, and *1-innermost* (see Materials and methods). Then we calculated the correlation coefficients between HC entropy and the 5-year survival rates in the three core levels (Fig 5). As depicted in the figure, the negative correlations between HC entropy and 5-year survival rates were preserved in all core levels identified by *R-core* decomposition. On the contrary, the *2-innermost* and *1-innermost* core levels identified by *K-core* decomposition showed positive correlations, which is significantly different from the negative correlation of whole network. In addition, we note that the average sizes (i.e., the average numbers of nodes) of *1*-*innermost* cores identified by *R-core* decomposition (Mean = 18.93; STDEV = 12.51) and *K-core* decomposition (Mean = 14.50; STDEV = 8.90) over the 16 cancer networks were not significantly different from each other (P = 0.290). Taken together, the innermost core identified by *R-core* decomposition better captures the relationship of HC entropy to cancer survivability than that of *K-core* decomposition, despite the observation that both decompositions identified *1-innermost* cores of similar size.

### Candidate cancer biomarker genes

Some recent studies reported that biomarker genes often reside in the innermost core of a biological network [18, 26, 27]. In addition, fragile genes, which are sensitive to replicative stress and exposure to environmental carcinogens, were also considered biomarkers in some cancers [51–53]. It has also been shown that a gene with a higher HC value is more likely to be a fragile gene in a human signaling network [28]. Guided by these results, we examined the three genes with the highest HC values in the *1*-*innermost* core as identified by *R*-*core* decomposition (Table 1). Interestingly, 33 genes out of a total of 48 genes were previously found to be biomarker genes for early cancer detection. For example, the three genes *KRAS*, *EGFR*, and *ERBB2*(*HER2*), which are found in the non-small cell lung cancer (NSCLC) signaling network, are key biomarkers [54, 55]. The *KRAS* gene is mutated in approximately 20% of NSCLC cases [56–58], and the *EGFR* gene is defective in approximately 10% of NSCLC patients and in nearly 50% of non-smoker lung cancer cases [59]. In addition, *HER2* mutations in NSCLC are present in approximately 4% of lung cancer patients [60]. Furthermore, the mutational frequency of the three genes may vary by race because of the impact of race/ethnicity on molecular pathways in human cancer [61, 62]]. For example, *EGFR* and *HER2* mutations among Korean lung cancer patients showed larger and lower frequencies, respectively, than that reported above [63]. Two additional genes, *CCND1* and *RB1*, identified in the bladder cancer signaling network are known biomarkers [64], and the third gene identified in the bladder cancer signaling network (*CDK4*) is a known inhibitor of bladder cancer [65, 66]. The top three genes found in the Glioma signaling network, *CCND1*, *CDKN2A*, and *RB1*, are frequently overexpressed, mutated, and/or deleted in glioma [67, 68]]. Molecular alterations of *EGFR* and *PIK3CA*, which are found in the endometrial cancer signaling network, have been reported in endometrial cancer studies [69, 70]. These examples suggest that the other top genes that have not been fully investigated yet may be promising candidate biomarkers.

**Table 1:** Candidate biomarker genes identified by *R-core* decomposition. In the table, **C1**, **C2**, and **C3** denote NCBI gene symbols of the three genes with the highest HC values among the *1-innermost* core genes as identified by *R-core* decomposition. The underlined genes mean they were previously reported as biomarker genes (see S1 Table).

Cancer site	Network properties				5-year survival rate	Candidate biomarker genes		
	The number of nodes	The number of edges	Degree entropy	HC entropy		C1	C2	C3
Acute myeloid leukemia	57	185	3.423	4.039	0.236	*FLT3*	*KIT*	*HRAS*
Basal cell carcinoma	47	534	2.588	2.646	0.914	*GLI1*	*GLI2*	*GLI3*
Bladder cancer	29	58	2.905	3.814	0.781	*RB1*	*CDK4*	*CCND1*
Breast cancer	144	773	3.905	4.138	0.897	*PGR*	*NCOA3*	*NCOA1*
Chronic myeloid leukemia	73	198	3.595	4.836	0.552	*CRK*	*CRKL*	*ABL1*
Colorectal cancer	49	153	3.365	4.002	0.636	*KRAS*	*PIK3R5*	*PIK3CA*
Endometrial cancer	44	121	2.884	3.845	0.686	*EGF*	*EGFR*	*PIK3CA*
Gastric cancer	148	682	3.506	4.824	0.306	*LRP5*	*LRP6*	*WNT16*
Glioma	64	255	3.719	4.437	0.334	*CCND1*	*CDKN2A*	*RB1*
Melanoma	68	719	2.853	3.148	0.912	*EGF*	*FGF1*	*FGF2*
Non-small-cell lung cancer	54	157	3.572	4.537	0.18	*KRAS*	*EGFR*	*ERBB2*
Pancreatic cancer	65	163	3.340	4.396	0.055	*KRAS*	*EGFR*	*JAK1*
Prostate cancer	86	404	3.836	4.371	0.994	*EGF*	*IGF1*	*INS*
Renal cell carcinoma	56	164	3.185	3.899	0.695	*HGF*	*MET*	*GAB1*
Small cell lung cancer	82	358	3.560	4.117	0.062	*ITGB1*	*LAMC3*	*COL4A1*
Thyroid cancer	28	73	2.601	3.267	0.972	*PPARG*	*PAX8*	*RXRA*

## Discussion

In this study, we investigated 16 types of cancer signaling networks and demonstrated that the hierarchical closeness entropy values and 5-year cancer survival rates were negatively correlated. This result suggested that when hierarchical closeness values of genes in a signaling network were more heterogeneous then these cancers are more likely incurable. SEER summaries of cancer survival rates do not account for cancer subtypes. Similarly, the KEGG pathways do not explicitly discern between cancer subtypes. For example, there are different subtypes in Melanoma cancer type, which have been characterized over the years: *BRAF* mutants [71, 72], *KRAS* mutants [73], *RAC1* mutants [74], *EGF* mutations [75], and other gene mutants. Patients affected by these subtypes have different survival times and respond differently to treatments. Therefore, we think that survival rate of a cancer patient is affected by two main factors: mutant position and signaling network structure. At a node level, mutations of a node at the innermost core are more deleteriously impactful than those at the rest of the network [17, 23, 76], thus such mutations may result in lower survival rates. At a network level, the network architecture with relatively high HC entropy results in lower cancer survival rates. The association between HC entropy and cancer survival rate at network structure level is a suggestion of prognostic model studies for cancer subtypes on the specific signaling pathways. Notably, the correlation between HC entropy and patient 5-year cancer survival rates was greater than the correlation between degree entropy and patient 5-year survival rates. In addition, HC entropy is a more attractive method than the modularity metric because of computational efficiency. Taken together, HC entropy can be considered an alternative to typical node-degree approaches for the associations between signaling network structure and cancer survivability.

To address the effects of HC entropy on patient 5-year survival rates, we simulated network robustness against mutations and found that the network robustness was positively correlated with the HC entropy of a network. This observation explains why cancer signaling networks with relatively high HC entropy are robust to therapeutic perturbations, which eventually make the cancer highly incurable. This result is also related to previous observations that showed highly modularized biological networks are sensitive to perturbations because distributions of HC values in these networks are likely to be relatively uniform [7, 50]. Two relationships (HC entropy, patient 5-year survival rate) and (HC entropy, network robustness) can be investigated further by a study of the correlation between patient 5-year survival rates and cancer network robustness.

Especially, we extended the reachability property to network decomposition and found that *R-core* decomposition is better than *K-core* decomposition in identifying cores that reflect the association between HC entropy and cancer survivability. In addition, it is known that cancer biomarker genes often reside at the innermost core in a biomolecule network. By highlighting examples of other known biomarkers, we showed that the genes with the highest HC values among the innermost *r*-core may be promising candidate cancer biomarkers. Altogether, the proposed approach may be useful in predicting the efficacy of cancer treatment and in identifying putative representative cancer genes.

## Supporting information

S1 Fig The distributions of HC values in pancreatic cancer and basal cell carcinoma signaling networks. (PDF)

S1 Table Previously reported cancer biomarker genes among the top three genes with the highest HC values in *1-innermost* R-core. (PDF)
