# Supplementary material for: Hierarchical closeness-based properties reveal cancer survivability and biomarker genes in molecular signaling networks
Source: PLoS One. 2018 Jun 18;13(6):e0199109. doi: 10.1371/journal.pone.0199109 (PMC6005509; doi:10.1371/journal.pone.0199109)
Supplement: S1 Fig — (PDF) [file pone.0199109.s001.pdf]

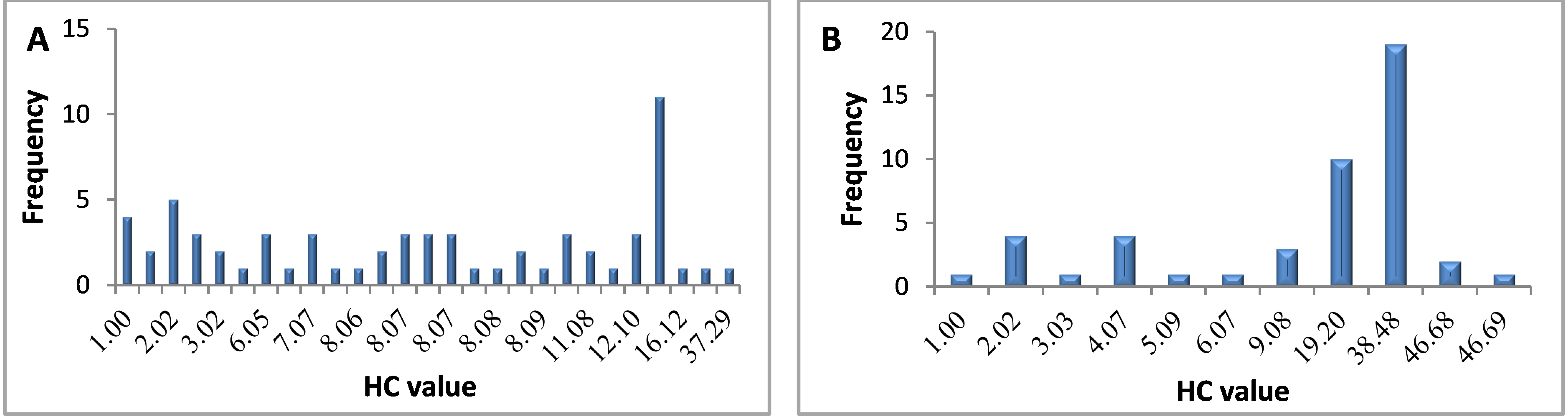

S1 Fig. The distributions of HC values in Pancreatic cancer and Basal cell carcinoma signaling networks. (A) Result of Pancreatic cancer signaling network. **(B)** Result of Basal cell carcinoma signaling network. Note that the reachability value and the closeness value are the integer and the decimal parts of a HC value, respectively.
