# Supplementary material for: Hierarchical closeness-based properties reveal cancer survivability and biomarker genes in molecular signaling networks
Source: PLoS One. 2018 Jun 18;13(6):e0199109. doi: 10.1371/journal.pone.0199109 (PMC6005509; doi:10.1371/journal.pone.0199109)
Supplement: S1 Table — (PDF) [file pone.0199109.s002.pdf]

S1 Table. Previously reported cancer biomarker genes among the top three genes with the highest HC values in*1-innermost* R-core.

Cancer site	Biomarker genes
Acute myeloid leukemia	*FLT3* [1]; *KIT* [2]
Basal cell carcinoma	*GLI1* [3]; *GLI2* [4], *GLI3* [5,6]
Bladder cancer	*RB1* [7]; *CCND1* [8,9]
Breast cancer	*PGR* [10]; *NCOA3* [11]; *NCOA1* [12]
Chronic myeloid leukemia	*CRKL* [13]; *ABL1* [14,15]
Colorectal cancer	*KRAS* [16,17]; *PIK3CA* [18]
Endometrial cancer	*EGFR* [19,20]; *PIK3CA* [20,21]
Gastric cancer	*LRP5* [22]; *WNT16* [23]
Glioma	*CCND1* [24]; *CDKN2A* [25]; *RB1* [26]
Melanoma	*EGF* [27]
Non-small-cell lung cancer	*KRAS* [28]; *EGFR* [29]; *ERBB2(HER2)* [30]
Pancreatic cancer	*KRAS* [31]; *EGFR* [32]
Prostate cancer	*EGF* [33]
Renal cell carcinoma	*HGF* [34]; MET [35]
Thyroid cancer	*PPARG* [36]; *PAX8* [37]

References

1. Fathi AT, Chen YB. Treatment of FLT3-ITD acute myeloid leukemia. American Journal of Blood Research. 2011;1(2):175–189.

2. Chen W, Xie H, Wang H, Chen L, Sun Y, Chen Z, et al. Prognostic Significance of KIT Mutations in Core-Binding Factor Acute Myeloid Leukemia: A Systematic Review and Meta-Analysis. PLOS ONE. 2016;11(1):e0146614.

3. Kuphal S, Shaw-Hallgren G, Eberl M, Karrer S, Aberger F, Bosserhoff AK, et al. GLI1-dependent transcriptional repression of CYLD in basal cell carcinoma. Oncogene. 2011;30(44):4523–4530.

4. Tojo M, Kiyosawa H, Iwatsuki K, Nakamura K, Kaneko F. Expression of the GLI2 oncogene and its isoforms in human basal cell carcinoma. Br J Dermatol. 2003;148(5):892–7.

5. Hutchin ME, Kariapper MST, Grachtchouk M, Wang A, Wei L, Cummings D, et al. Sustained Hedgehog signaling is required for basal cell carcinoma proliferation and survival: conditional skin tumorigenesis recapitulates the hair growth cycle. Genes & Development. 2005;19(2):214–223.

6. Epstein EH. Basal cell carcinomas: attack of the hedgehog. Nature reviews Cancer. 2008;8(10):743–754.

7. Yin M, Grivas P, Ali SM, Hsu J, Vasekar MK, Emamekhoo H, et al. ATM/RB1 mutations to predict shorter overall survival (OS) in bladder cancer. Journal of Clinical Oncology. 2017;35(6_suppl):393–393.

8. Netto GJ. Molecular biomarkers in urothelial carcinoma of the bladder: are we there yet? Nat Rev Urol. 2012;9(1):41–51.

9. Seiler R, Thalmann GN, Rotzer D, Perren A, Fleischmann A. CCND1/CyclinD1 status in metastasizing bladder cancer: a prognosticator and predictor of chemotherapeutic response. Mod Pathol. 2014;27(1):87–95.

10. Patil AV, Bhamre RS, Singhai R, Tayade MB, Patil VW. Estrogen receptor (ER) and progesterone receptor (PgR) in breast cancer of Indian women. Breast Cancer : Targets and Therapy. 2011;3:27–33.

11. Burwinkel B, Wirtenberger M, Klaes R, Schmutzler RK, Grzybowska E, Försti A, et al. Association of NCOA3 Polymorphisms with Breast Cancer Risk. Clinical Cancer Research. 2005;11(6):2169–2174. doi:10.1158/1078-0432.CCR-04-1621.

12. Qin L, Wu YL, Toneff MJ, Li D, Liao L, Gao X, et al. NCOA1 Directly Targets M-CSF1 Expression to Promote Breast Cancer Metastasis. Cancer Research. 2014;74(13):3477–3488. doi:10.1158/0008-5472.CAN-13-2639.

13. Nichols GL, Raines MA, Vera JC, Lacomis L, Tempst P, Golde DW. Identification of CRKL as the constitutively phosphorylated 39-kD tyrosine phosphoprotein in chronic myelogenous leukemia cells. Blood. 1994;84(9):2912–8.

14. Goldman JM, Melo JV. BCR-ABL in chronic myelogenous leukemia–how does it work? Acta Haematol. 2008;119(4):212–7.

15. Giri S, Pathak R, Martin MG, Bhatt VR. Characteristics and survival of BCR/ABL negative chronic myeloid leukemia: a retrospective analysis of the Surveillance, Epidemiology and End Results database. Therapeutic Advances in Hematology. 2015;6(6):308–312.

16. Yokota T. Are KRAS/BRAF Mutations Potent Prognostic and/or Predictive Biomarkers in Colorectal Cancers? Anti-Cancer Agents in Medicinal Chemistry. 2012;12(2):163–171.

17. Larki P, Gharib E, Yaghoob Taleghani M, Khorshidi F, Nazemalhosseini-Mojarad E, Asadzadeh Aghdaei H. Coexistence of KRAS and BRAF Mutations in Colorectal Cancer: A Case Report Supporting The Concept of Tumoral Heterogeneity. Cell Journal (Yakhteh). 2017;19(Suppl 1):113–117.

18. Cathomas G. PIK3CA in Colorectal Cancer. Frontiers in Oncology. 2014;4:35.

19. Khalifa MA, Abdoh AA, Mannel RS, Haraway SD, Walker JL, Min KW. Prognostic utility of epidermal growth factor receptor overexpression in endometrial adenocarcinoma. Cancer. 1994;73(2):370–376.

20. Hayes MP, Douglas W, Ellenson LH. Molecular alterations of EGFR and PIK3CA in uterine serous carcinoma. Gynecologic Oncology. 2009;113(3):370–373.

21. Konstantinova D, Kaneva R, Dimitrov R, Savov A, Ivanov S, Dyankova T, et al. Rare mutations in the PIK3CA gene contribute to aggressive endometrial cancer. DNA Cell Biol. 2010;29(2):65–70.

22. Liu X, Huang MZ, Chen ZY, Zhao XY, Wang CC, Peng W, et al. LRP5 polymorphism—A potential predictor of the clinical outcome in advanced gastric cancer patients treated with EOF regimen. Chinese Journal of Cancer Research. 2014;26(4):478–485.

23. Norollahi SE, Alipour M, Rashidy-Pour A, Samadani AA, Larijani LV. Regulatory Fluctuation of WNT16 Gene Expression Is Associated with Human Gastric Adenocarcinoma. Journal of Gastrointestinal Cancer. 2017;doi:10.1007/s12029-017-0022-y.

24. Büschges R, Weber RG, Actor B, Lichter P, Collins VP, Reifenberger G. Amplification and Expression of Cyclin D Genes (CCND1 CCND2 and CCND3) in Human Malignant Gliomas. Brain Pathology. 1999;9(3):435–442.

25. Ishii N, Maier D, Merlo A, Tada M, Sawamura Y, Diserens AC, et al. Frequent co-alterations of TP53, p16/CDKN2A, p14ARF, PTEN tumor suppressor genes in human glioma cell lines. Brain Pathol. 1999;9(3):469–79.

26. Bäcklund, L. Magnus and Nilsson, Bo R. and Goike, Helena M. and Schmidt, et al. Short Postoperative Survival for Glioblastoma Patients with a Dysfunctional Rb1 Pathway in Combination with No Wild-type PTEN. American Association for Cancer Research. 2003;11(9):4151–4158.

27. McCarron SL, Bateman AC, Theaker JM, Howell WM. EGF +61 gene polymorphism and susceptibility to and prognostic markers in cutaneous malignant melanoma. Int J Cancer. 2003;107(4):673–5.

28. Dumenil C, Vieira T, Rouleau E, Antoine M, Duruisseaux M, Poulot V, et al. Is there a specific phenotype associated with the different subtypes of KRAS mutations in patients with advanced non-small-cell lung cancers? Lung Cancer. 2015;90(3):561–7.

29. Rosell R, Moran T, Queralt C, Porta R, Cardenal F, Camps C, et al. Screening for Epidermal Growth Factor Receptor Mutations in Lung Cancer. New England Journal of Medicine. 2009;361(10):958–967.

30. Garrido-Castro AC, Felip E. HER2 driven non-small cell lung cancer (NSCLC): potential therapeutic approaches. Translational Lung Cancer Research. 2013;2(2):122–127.

31. Tao Ly, Zhang Lf, Xiu Dr, Yuan Ch, Ma Zl, Jiang B. Prognostic significance of K-ras mutations in pancreatic cancer: a meta-analysis. World Journal of Surgical Oncology. 2016;14(1):146.

32. Li Q, Zhang L, Li X, Yan H, Yang L, Li Y, et al. The prognostic significance of human epidermal growth factor receptor family protein expression in operable pancreatic cancer. BMC Cancer. 2016;16(1):910.

33. Bhat FA, Sharmila G, Balakrishnan S, Singh PR, Srinivasan N, Arunakaran J. Epidermal growth factor-induced prostate cancer (PC3) cell survival and proliferation is inhibited by quercetin, a plant flavonoid through apoptotic machinery. Biomedicine & Preventive Nutrition. 2014;4(4):459–468.

34. Tanimoto S, Fukumori T, El-Moula G, Shiirevnyamba A, Kinouchi S, Koizumi T, et al. Prognostic significance of serum hepatocyte growth factor in clear cell renal cell carcinoma: comparison with serum vascular endothelial growth factor. J Med Invest. 2008;55(1-2):106–11.

35. Macher-Goeppinger S, Keith M, Endris V, Penzel R, Tagscherer KE, Pahernik S, et al. MET expression and copy number status in clear-cell renal cell carcinoma: prognostic value and potential predictive marker. Oncotarget. 2017;8(1):1046–1057.

36. Algeciras-Schimnich A, Milosevic D, McIver B, et al. Evaluation of the PAX8/PPARG Translocation in Follicular Thyroid Cancer with a 4-Color Reverse-Transcription PCR Assay and Automated High-Resolution Fragment Analysis. Clinical chemistry. 2010;56(3):391-398.

37. Raman P, Koenig RJ. PAX8-PPARγ fusion protein in thyroid carcinoma. Nature reviews Endocrinology. 2014;10(10):616–623.
